# An Evaluation of the Hitit Index in Differential Diagnosis of Crimean-Congo Hemorrhagic Fever in the Emergency Department

**DOI:** 10.3390/medicina59101796

**Published:** 2023-10-09

**Authors:** Seval Komut, Nurullah Çorakyer, Gülcan Kaplan, Nurcan Baykam

**Affiliations:** 1 Department of Emergency Medicine, Faculty of Medicine, Hitit University, 19040 Çorum, Turkey; nurullah.corakyer@saglik.gov.tr; 2 Department of Infectious Diseases and Clinical Microbiology, Faculty of Medicine, Hitit University, 19040 Çorum, Turkey; gulcankaplan@hitit.edu.tr (G.K.); nurcanbaykam@hitit.edu.tr (N.B.)

**Keywords:** emergency department, Crimean-Congo hemorrhagic fever, Hitit index

## Abstract

*Background and Objectives*: Crimean-Congo Hemorrhagic Fever (CCHF) is a viral zoonotic infection, which is seen over a wide geographic area. The mortality rate is in inverse proportion to the ability of patients to access healthcare services. Therefore, early identification of patients is extremely important. The aim of this study was to test the sensitivity and specificity of the Hitit Index in the differentiation of CCHF cases at the time of presentation at the Emergency Department and to evaluate the agreement of this index with molecular (CCHFV RNA) and/or serological diagnostic tests (ELISA-CCHF IgM). *Materials and Methods:* The patients included were those who presented at the Emergency Department (ED) with the complaint of a tick bite or those identified as potential CCHF cases as a result of complaints and/or laboratory findings. For cases that met the study inclusion criteria, the Hitit Index score was calculated automatically from the parameters included in the index formula uploaded to the automation system in the ED at the time of presentation. Through comparisons of the agreement of the Hitit Index with the CCHFV-RNA and/or IgM results the power of the Hitit Index for differentiation of CCHF cases in ED was evaluated. *Results:* The data of 273 patients were analyzed. There was a history of tick bite in 236 (86%) cases. Of the evaluated cases, 110 (40.2%) were hospitalized; CCHF positivity was determined in 72 (26.4%). The Hitit Index values calculated in ED and at 24 h after hospitalization were determined to be significant in the prediction of the CCHF cases (*p* < 0.001, AUC = 0.919 (0.887–0.951); *p* < 0.001, AUC = 0.902 (0.841–0.962). For a cut-off point of 0 of the Hitit Index evaluated in ED, the classification success was found to have a sensitivity of 75% and specificity of 88% (PPV-NPV). For a cut-off point of 0 of the Hitit Index evaluated at 24 h after hospitalization, the classification success was found to have a sensitivity of 79.7% and specificity of 84% (PPV-NPV). *Conclusions:* The defined form of the Hitit Index can be used in the differentiation of CCHF cases in ED with high sensitivity and specificity levels. Just as evaluation with the Hitit Index prevents unnecessary hospitalization, it can also contribute to reducing mortality rates with the early identification of CCHF cases.

## 1. Introduction

Crimean-Congo Hemorrhagic Fever (CCHF) is a viral zoonotic infection, which is seen over a wide geographic area [1]. The virus was first isolated in an outbreak in Crimea in 1944, and following determination of the same strain isolated from a patient in the Belgian Congo in 1956, it was named Crimean-Congo Hemorrhagic Fever virus [2]. CCHF virus is a membranous RNA virus of the genus Nairuvirus from the Bunyavirales family [3,4,5]. Ticks of the *Hyalomma* species, especially *H. marginatum* are the primary vector and reservoir of the virus. The disease is seen with many different presentations, from an asymptomatic form to a severe form progressing with bleeding. The mortality rate of the disease varies from 5% to 30% and has been reported to be 4–5% in Turkey [4,6].

The mortality rate varies in inverse proportion to the ability of patients to access healthcare services and the degree to which the healthcare services of the country are developed. Therefore, early identification of patients and access to the necessary medical care are extremely important. A definitive diagnosis of the disease is made from the determination of CCHFV RNA and/or virus-specific antibodies with polymerized chain reaction (PCR) and ELISA tests. However, these tests are not available in every hospital, so patient samples are sent to reference laboratories [7]. According to the algorithm in the reference laboratory, the first samples which were sent from possible acute cases were searched initially by an in-house real-time PCR method and those which were found negative with PCR were then studied by the in-house ELISA method in terms of CCHF-IgM antibodies [8].

As a certain time is required for test results, there is a need for an easily accessible scoring system that can be used primarily in emergency departments and polyclinic conditions to provide the correct selection of potential patients for early hospitalization.

In 2019, the Hitit Index was introduced by the Infectious Diseases and Clinical Microbiology Clinics of Hitit University Medical Faculty, as an index formed from clinical symptoms and basic laboratory tests to define CCHFV infection [7].

The aim of this study was to test the sensitivity and specificity of the Hitit Index in the differentiation of CCHF cases, and in the provision of early hospitalization, in cases suspected of CCHF at the time of presentation at the Emergency Department and to evaluate the agreement of this index with serological (ELISA-CCHF IgM) and molecular diagnostic tests (CCHFV-RNA).

## 2. Materials and Methods

Approval for the study was granted by the Local Ethics Committee (decision no: 2022-99, dated: 30 November 2022). The study was conducted in the Emergency Medicine and Infectious Diseases and Clinical Microbiology Clinics of Erol Olçok Training and Research Hospital. The patients included were those who presented at the Emergency Department (ED) between March and October 2022 with the complaint of a tick bite, which is one of the most well-known routes of transmission of CCHF, or those identified as potential cases as a result of complaints and/or laboratory findings.

Identification of potential cases: As Çorum, where the study was conducted, is an endemic region for CCHF, cases that met the clinical definition and had at least two supporting findings were accepted as potential CCHF [9] (Table 1).

Leukopenia, thrombocytopenia, elevated aspartate aminotransferase (AST), alanine aminotransferase (ALT), lactate dehydrogenase (LDH), and creatinine kinase (CK) determined in laboratory tests were accepted as abnormal and suspicious findings.

For cases that met the study inclusion criteria, the Hitit Index score was calculated automatically from the parameters included in the index formula uploaded to the automation system in the ED at the time of presentation. The International Normalized Ratio (INR), fibrinogen, direct bilirubin, AST, CK, hematocrit (HTC), Glomerular Filtration Rate (GFR) levels, and the lymphocyte, neutrophil, and red blood cell (RBC) counts, as well as the presence of conjunctival hyperemia were used in the calculation of the Hitit Index. These data were followed up prospectively. The decision on the hospitalization of patients evaluated in ED was made by an Infectious Diseases and Clinical Microbiology clinician evaluating clinical and laboratory findings and the Hitit Index results together. Through comparisons of the agreement of the Hitit Index with the CCHF-RNA PCR and CCHF IgM results of the cases that were hospitalized and followed up, the power of the Hitit Index for differentiation of CCHF cases in ED was evaluated.

Since CCHFV PCR positivity was detected in the first 9 days of the disease, and IgM positivity developed later, the diagnosis of the patients was made by investigating the CCHFV-PCR positivity by an in-house real-time PCR method in serum samples obtained on the day of hospitalization. In cases where PCR positivity was not detected, the diagnosis was made by demonstrating CCHF-IgM antibodies with an in-house ELISA method. PCR or IgM positivity is sufficient in the diagnostic algorithm. For this reason, IgG positivity or a 4-fold increase in IgG titer was not investigated in cases where PCR or ELISA positivity was detected.

Hitit Index Formula: 5.6 − (5.3 × lymphocyte) − (0.02 × fibrinogen) − (12 × direct bilirubin) + (0.04 × AST) + (0.32 × hematocrit) − (0.5 × neutrophil) − (0.07 × CKD − EPI) − (0.001 × CK) ± conjunctival hyperemia (+1.5 in conjunctival hyperemia presence and −1.5 in conjunctival hyperemia absence) [5].

AST: Aspartate aminotransferase, CKD-EPI: Chronic Kidney Disease Epidemiology Collaboration, CK: Creatine Kinase.

### Statistical Analysis

The data were analyzed statistically using SPSS version 22 software (SPSS Inc., Chicago, IL, USA). The Shapiro–Wilk test was used to determine whether the data were normally distributed. Depending on the data distribution, descriptive statistics of numerical data were reported as mean ± standard deviation or median (min-max) values. Categorical variables were stated as number (*n*) and percentage (%). The Mann–Whitney U-test was used to compare non-normally distributed numerical data between two independent groups. Depending on the sample sizes in the crosstab cells, proportional comparisons between categorical variables were performed using the Chi-square test.

ROC (Receiver Operating Characteristic) analysis was used to determine whether clinical and laboratory values are prognostic indicators for CCHF prediction. The ROC area under the curve (AUC) with 95% confidence intervals was computed. The AUC values obtained as a result of the ROC analyses were interpreted as follows: 0.9–1: excellent, 0.8–0.9: good, 0.7–0.8: fair, 0.6–0.7: poor, and 0.5–0.6: unsuccessful. Following the ROC analysis, the best cut-off point for the Hitit Index in CCHF estimation was determined using the Youden index (maximum sensitivity and specificity). Sensitivity, specificity, accuracy, PPV (positive predictive value), NPV (negative predictive value), and positive likelihood ratio (LR+) values were used to assess the success of the cut-off points determined for the Hitit index in CCHF estimation. The level of statistical significance level was accepted as *p* < 0.05.

## 3. Results

The data of 273 patients were analyzed. All these cases evaluated in ED lived in the province of Çorum, which is an endemic region. There was a history of tick bite in 236 (86%) cases. Of the evaluated cases, 110 (40.2%) were hospitalized. Blood samples of only the hospitalized cases were sent to the reference laboratory for CCHF testing and, of these, CCHF positivity was determined in 72 (26.4%).

Patients who were not thought to have CCHF by evaluation of the clinical findings, laboratory tests, and Hitit Index in ED (*n*: 164, 60.1%) were discharged home and instructed to present again at ED when necessary to identify disease findings. Whether or not these cases presented again, at the ED, polyclinics, or other centers, with findings consistent with CCHF was followed up by the healthcare network system. Cases with no subsequent presentation were evaluated in the category of CCHF-negative cases together with PCR-negative cases *(n*: 201, 73.6%). The mean age of all the patients included in the study was 45.32 ± 16.49 years (range, 18–80 years) and the mean age of the CCHF-positive patients was 51.67 ± 16.94 years (range, 19–79 years).

The statistical results of the comparisons between the laboratory blood values and the clinical characteristics of the patients diagnosed with CCHF and those in which CCHF was excluded are presented in Table 2.

The direct bilirubin, AST, and CK values of the CCHF cases were found to be significantly high (*p* < 0.001 for all) (Table 2). The lymphocyte, HTC, and neutrophil values of the CCHF cases were determined to be significantly low (*p* < 0.001, *p* = 0.020, *p* < 0.001) (Table 2). No statistically significant difference was determined between the groups in respect of INR, fibrinogen, CKD-EPI, and RBC values (*p* > 0.05) (Table 2).

Conjunctival hyperemia was seen to be predominant as a significant finding in CCHF cases (*p* = 0.002) (Table 2).

The sensitivity, specificity, accuracy, positive–negative predictive values, and likelihood ratio (+) calculated using the cut-off values resulting from the ROC analysis performed to determine the success rates of PCR prediction for different cut-off values of the Hitit Index calculated in ED and at 24 h after hospitalization, are presented in Table 3. According to the results of the ROC analysis, the Hitit Index values calculated in ED and at 24 h after hospitalization were determined to be significant in the prediction of the CCHF PCR result (*p* < 0.001, AUC = 0.919 (0.887–0.951); *p* < 0.001, AUC = 0.902 (0.841–0.962) (Table 3).

For a cut-off point of 0 of the Hitit Index evaluated in ED, the classification success was found to have a sensitivity of 75% and specificity of 88%. Using the Youden Index, an alternative cut-off point for the Hitit Index was determined as −3.55, with 91.6% sensitivity and 80.5% specificity.

**Figure 1:**
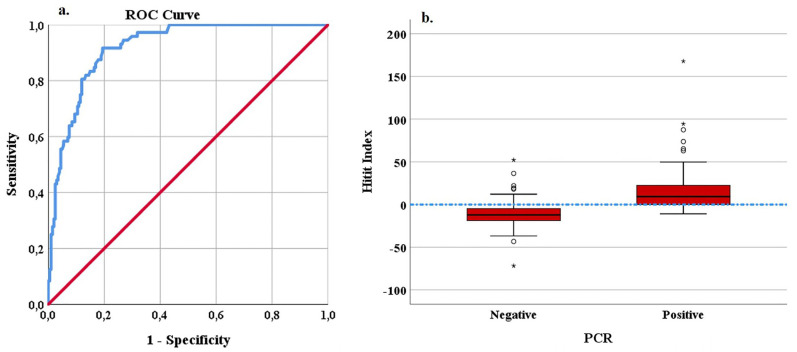
ROC curve (**a**) and box plot (**b**) of the Hitit Index in the prediction of CCHF-PCR. In a box plot graph, observations that fall outside the boundaries of Q1 − 3xIQR and Q3 + 3xIQR are defined as extreme values and are indicated with “*”. (IQR = Q3 − Q1).

**Figure 2:**
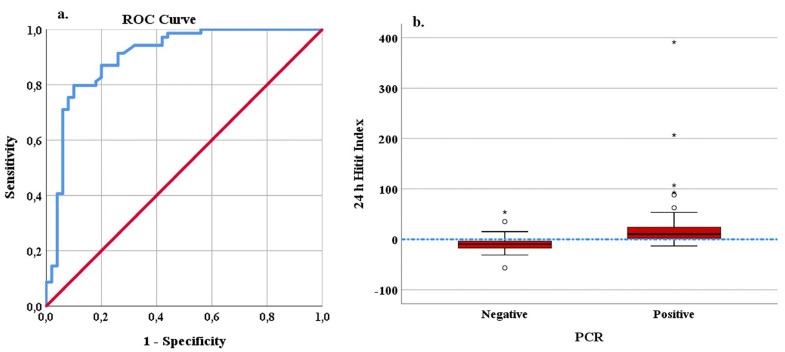
ROC curve (**a**) and box plot (**b**) of the 24 h Hitit Index in the prediction of CCHF-PCR. In a box plot graph, observations that fall outside the boundaries of Q1 − 3xIQR and Q3 + 3xIQR are defined as extreme values and are indicated with “*”. (IQR = Q3 − Q1).

For a cut-off point of 0 of the Hitit Index evaluated 24 h after hospitalization, the classification success was found to have a sensitivity of 79.7% and specificity of 84%. Using the Youden Index, an alternative cut-off point for the Hitit Index was determined to be 0.85, with no change in sensitivity (79.7%) and specificity was observed to have increased to 90%. For this cut-off point, increases in PPV to 91.6% and NPV to 76.2% were determined. The ROC curve and box graph obtained for the prediction of CCHF using the Hitit Index in the ED are shown in Figure 1, and the ROC curve and box graph obtained for prediction after 24 h are shown in Figure 2.

The findings showing the correct and incorrect categorization of the Hitit Index calculated in ED and after 24 h of hospitalization according to the different cut-off values, determined as a result of the ROC analysis performed to predict the CCHF result, are shown in Table 4.

## 4. Discussion

CCHFV is found in animals, such as cattle, sheep, goats, rabbits, and foxes, without causing disease. Ticks of the Hyalomma species, especially *H. marginatum* are the primary vector and reservoir of the virus. Other than Hyalomma, it has also been shown that ticks of Dermacentor marginatus, Rhipicephalus rossicus, and Amblyomma variegatum species can carry the virus [10]. Transmission of the virus to humans is through direct contact with infected animals, via the blood, tissue, or bodily fluids of infected patients, or from bites from infected ticks [11]. Therefore, the global distribution of CCHFV is associated with the distribution of Hyalomma species ticks. However, only 50–60% of patients have a history of tick contact. The absence of tick contact is not sufficient to discount the disease. Shepherds, farmers, others engaged in animal husbandry, slaughterhouse workers, family members caring for patients, and healthcare workers are at risk of disease [12,13].

CCHF is endemic in countries south of the 50th north parallel latitude in Africa, the Balkans, the Middle East, and Asia [14]. The vast majority of CCHF cases seen in Turkey have been reported from the north of Central Anatolia, Central Black Sea, and Eastern Anatolia regions. The current study was conducted in the province of Çorum, in the north of Central Anatolia, which is a region where the disease is seen to be endemic, and CCHF cases are reported each year in the spring and summer months. Our hospital is in a central location, admitting, treating, and following up CCHF cases from the center of Çorum and the surrounding provincial towns [15].

The disease can show an asymptomatic or subclinical course in some cases. In endemic regions, asymptomatic cases have been reported at rates varying from 27% to 88% [16,17,18]. Four phases of the disease have been identified. The incubation period lasts for 1–3 days (maximum 9 days) and, at the end of this period, symptomatic cases enter the pre-hemorrhagic phase. Patients in the pre-hemorrhagic phase present at ED and polyclinics with symptoms such as fever, headache, dizziness, myalgia, nausea, vomiting, and diarrhea, as well as findings of conjunctival and facial hyperemia [16,17,19]. While some patients remain in this phase and enter a convalescence period, some enter the hemorrhagic phase as a result of vascular pathologies, coagulation disorder, and disseminated intravascular coagulation (DIC) caused by cytokine storm triggered by infection. In the hemorrhagic phase, petechiae, ecchymosis, hematoma, gingival and nasal bleeding, hematuria, vaginal bleeding, hematemesis, and melena occur.

Some cases with a severe course are lost in this phase. The mortality rate has been reported to increase to 30–40% in different series [14]. In countries such as Turkey, where healthcare services can be accessed rapidly and relatively easily, the mortality rate is around 5%, as healthcare is better. However, even this rate is above the acceptable rate for an infectious disease. One of the ways to lower the mortality rates is to identify and closely follow up cases at the start of the pre-hemorrhagic phase in ED and under polyclinic conditions. The determination of leukopenia, thrombocytopenia, and elevated AST, ALT, LDH, and CK, in the hemogram and biochemical tests requested in ED and polyclinics, facilitates the differentiation of suspected CCHF cases [20].

The Ministry of Health of the Republic of Turkey has defined potential and definite cases based on the evaluation of epidemiological data, clinical findings, and laboratory test results. A definitive diagnosis of the disease is based on the determination of CCHFV-PCR positivity in the first 10 days or on the serological determination of CCHFV IgM antibodies (Table 1) [7]. However, because of the intensity of the disease in certain regions of the country and the high testing costs, these tests are examined collectively in regional reference laboratories. This causes a delay in diagnosis. Therefore, there is a need for the development of different approaches to be able to immediately identify the test results of cases living in an endemic region, with or without a history of tick bite, who present at the ED, especially in the early stage of the disease.

The Hitit Index, defined in 2019 by the Medical Faculty of Hitit University, was formed from the evaluation of some of the clinical and laboratory results on the first day of hospitalization of 65 cases hospitalized with an initial diagnosis of CCHF in 2018 [7]. When a cut-off value of 0 was taken in that study, the accuracy, sensitivity, specificity, PPV, and NPV values of the index for the differentiation of CCHF positive and negative cases were reported to be extremely high (92%, 96%, 90%, 87%, and 97%, respectively).

The aim of the current study was to measure the power of the Hitit Index in predicting CCHF cases presenting at ED in the early phase of the disease by comparing the parameters in the Hitit Index between the CCHF-positive and negative cases. In the group with a confirmed CCHF diagnosis, the direct bilirubin, AST, and CK values were significantly elevated, and lymphopenia and neutropenia were seen to be consistent with the first study in which the Hitit Index was defined [7]. The elevated hematocrit level, which was significant in the multivariate logistic regression analysis in CCHF-positive patients in the first study and was reported to reduce false positivity in the Hitit Index, was determined to be significantly low in the current study. Low fibrinogen, one of the parameters determining disease severity, was not determined in the patients presenting at ED in the early phase, and no difference was determined between the CCHF-positive and negative patients with respect to the INR value. In the first study, which defined the Hitit Index, it was emphasized that the median CKD-EPI value was significantly low and that this could be the source of intrarenal hemodynamic dysregulation caused by endothelial dysfunction and cytokine storm in CCHF has been supported in the literature [7,21,22,23]. However, in the current study, no significant difference was determined between the two groups with respect to the CKD-EPI values. The presence of conjunctival hyperemia, which has previously been reported as the only clinical finding with statistical significance in CCHF cases, was found to be significant in the current study.

The sensitivity and specificity values of the Hitit Index score were found to be similar when calculated in ED for the 273 cases and at 24 h after hospitalization for the 110 hospitalized patients (75% and 88–79.7% and 84%). According to the ROC analysis results, the AUC values of both the Hitit Index in ED and the Hitit Index at 24 h were determined to be >0.90, indicating an excellent level. Therefore, it was concluded that the Hitit Index was significant in the prediction of CCHF cases and was suitable for use in ED for this purpose.

When alternative cut-off values were determined using the Youden Index, the cut-off value reduced from 0 to −3.55 in ED, increasing sensitivity and slightly reducing specificity (91.6% and 80.5%). With the alternative cut-off value of 0.85 after 24 h, sensitivity did not change but specificity increased (79.7% and 90%) [7]. As a result of the calculations made using the new cut-off values, the sensitivity and specificity rates were determined to increase, and, by adding different laboratory test results and clinical parameters to the index and trialing different cut-off values, it was seen that the index could be improved.

## 5. Conclusions

Early diagnosis and treatment are extremely important for CCHF cases. The defined form of the Hitit Index can be used in the differentiation of CCHF cases in ED with high sensitivity and specificity levels. Just as evaluation with the Hitit Index prevents unnecessary hospitalization, it can also contribute to reducing mortality rates with the early identification of CCHF cases and the provision of healthcare services. As transmission occurs through contact and tick attachment, a certain number of cases are encountered each year in endemic regions and it is not possible to reach case series which will allow prospective studies of larger series. Therefore, the formation of new models should be attempted to increase the sensitivity and specificity of the index with a greater number of cases, which could be conducted prospectively in the ED in the next few years.

## Figures and Tables

**Table 1:** The Ministry of Health of the Republic of Turkey definitions of CCHF cases.

**Clinical Definition**
At least 2 of the following 4 clinical criteria: 1. At least 2 of the following complaints: Fever (≥38 °C) Listlessness Headache Generalised body pain Joint pain Diarrhea 2. Findings of bleeding of the skin and mucosa 3. Thrombocytopenia and/or leukopenia which cannot be explained by another reason 4. Elevated ALT and AST which cannot be explained by another reason
**Epidemiological Criteria**
Within the 2 weeks prior to disease onset: 1. A history of tick contact or attachment 2. A history of contact with animal blood, tissue, or secretions 3. Living in a rural area or history of travel to a rural area 4. A history of close contact with a diagnosed case
**Laboratory Criteria**
1. Virus isolation 2. Determination of virus-specific IgM antibody positivity 3. Determination of an increase of >4 fold in virus-specific IgG titer in the serum in the acute and convalescent periods 4. Determination of viral nucleic acid

**Potential Case:** case that meets the clinical definition and meets at least one of the epidemiological criteria. **Confirmed Case:** potential case confirmed with at least one of the laboratory criteria.

**Table 2:** Comparison of laboratory blood values and clinical characteristics among diagnosed-with-CCHF and CCHF-excluded groups.

		Diagnostic Status for CCHF		
		CCHF Excluded (*n* = 201)	Diagnosed with CCHF (*n* = 72)	*p*-Values
INR		1.06 ± 0.10 1.04 (0.88–1.78)	1.11 ± 0.22 1.07 (0.09–1.64)	0.095 ^a^
Fibrinogen		266.3 ± 119.2 256 (110–684)	251.8 ± 78.12 234.5 (105–457)	0.563 ^a^
Direct Bilirubin		0.12 ± 0.10 0.11 (0.03–1.23)	0.16 ± 0.10 0.13 (0.04–0.58)	**<0.001 ^a^**
AST		33.83 ± 56.61 22 (10–544)	206.8 ± 275.5 110.5 (1–1776)	**<0.001 ^a^**
CK		244.5 ± 888.3 122 (0–12228)	682.8 ± 1044 231 (21–4653)	**<0.001 ^a^**
CKDEPI		92 ± 24.14 95 (6–156)	92.35 ± 24.92 93 (28–145)	0.764
Lymphocyte		2.24 ± 1.04 2.20 (0.22–5.99)	0.68 ± 0.39 0.56 (0.16–1.82)	**<0.001 ^a^**
HTC		40.86 ± 4.80 41.3 (14.7–50.2)	39.32 ± 6.07 39.65 (13.4–54.2)	**0.020 ^a^**
Neutrophil		4.68 ± 2.29 4.31 (1.27–20.97)	2.17 ± 2.19 1.4 (0.34–11.91)	**<0.001 ^a^**
RBC		4.79 ± 0.6 4.79 (1.02–7)	4.77 ± 0.61 4.74 (3.22–6.70)	0.601 ^a^
Conjunctival Hyperemia	No	167 (83.1%)	47 (65.3%)	**0.002 ^b^**
	Yes	34 (16.9%)	25 (34.7%)	

^a^ Mann–Whitney U test with mean ± standard deviation and median (min-max). ^b^ Chi-Square test with *n* (%). INR: International Normalized Ratio, AST: Aspartate Aminotransferase, CK: Creatine Kinase, CKDEPI: Chronic Kidney Disease Epidemiology Collaboration, HTC: Hematocrit, RBC: Red Blood Cell.

**Table 3:** Sensitivity, specificity, PPV, NPV, and likelihood ratio (+) values of Hitit Index and 24 h Hitit Index in the prediction of CCHF-PCR.

	Hitit Index (*n* = 273)	Hitit Index (*n* = 273)	24 h Hitit Index (*n* = 119)	24 h Hitit Index (*n* = 119)
Cut off	≥0	≥−3.55	≥0	≥0.85
AUC	0.919 (0.887–0.951)	0.919 (0.887–0.951)	0.902 (0.841–0.962)	0.902 (0.841–0.962)
Sensitivity (95% CI)	0.75 (0.631–0.841)	0.916 (0.821–0.965)	0.797 (0.679–0.880)	0.797 (0.679–0.880)
Specificity (95% CI)	0.880 (0.825–0.920)	0.805 (0.743–0.856)	0.84 (0.703–0.923)	0.9 (0.774–0.962)
Accuracy	0.846	0.835	0.815	0.840
PPV (95% CI)	0.692 (0.576–0.789)	0.628 (0.528–0.719)	0.873 (0.759–0.939)	0.916 (0.808–0.968)
NPV (95% CI)	0.907 (0.855–0.942)	0.964 (0.920–0.985)	0.75 (0.613–0.851)	0.762 (0.631–0.859)
LR+ (95% CI)	6.28 (4.21–9.35)	4.72 (3.53–6.31)	4.98 (2.61–9.50)	7.97 (3.44–18.46)

CCHF: Crimean-Congo Hemorrhagic Fever, CI: Confidence Interval, AUC: Area Under Curve, PPV: Positive Predictive Value, NPV: Negative Predictive Value, LR+: Probability that a person with the disease tested positive/probability that a person without the disease tested positive.

**Table 4:** The success of cut-off values, determined by ROC analysis, in CCHF-PCR prediction.

	Cut-Off	PCR		Total
		Negative	Positive	
Hitit Index	Negative (<0)	177	18	195
	Positive (≥0)	24	54	78
Hitit Index	Negative (<−3.55)	162	6	168
	Positive (≥−3.55)	39	66	105
Total		201	72	273
24 h Hitit Index	Negative (<0)	42	14	56
	Positive (≥0)	8	55	63
24 h Hitit Index	Negative (<0.85)	45	14	59
	Positive (≥0.85)	5	55	60
Total		50	69	119

## Data Availability

All data generated or analyzed during this study are included in this published article.
